# Uses, traditional management, perception of variation and preferences in ackee (*Blighia sapida *K.D. Koenig) fruit traits in Benin: implications for domestication and conservation

**DOI:** 10.1186/1746-4269-6-12

**Published:** 2010-03-19

**Authors:** Marius RM Ekué, Brice Sinsin, Oscar Eyog-Matig, Reiner Finkeldey

**Affiliations:** 1 Forest Genetics and Forest Tree Breeding, Büsgen-Institute, Georg-August University of Göttingen, Büsgenweg 2, 37077 Göttingen, Germany; 2 Laboratoire d'Ecologie Appliquée, Faculté des Sciences Agronomiques, Université d'Abomey-Calavi, 01 BP 526 Cotonou, Bénin; 3 Sub-Saharan African Forest Genetic Resources Programme, Bioversity International c/o CIFOR Regional Office In Cameroon PO Box 2008 Messa, Yaounde, Cameroon

## Abstract

**Background:**

*Blighia sapida *is a woody perennial multipurpose fruit tree species native to the Guinean forests of West Africa. The fleshy arils of the ripened fruits are edible. Seeds and capsules of the fruits are used for soap-making and all parts of the tree have medicinal properties. Although so far overlooked by researchers in the region, the tree is highly valued by farmers and is an important component of traditional agroforestry systems in Benin. Fresh arils, dried arils and soap are traded in local and regional markets in Benin providing substantial revenues for farmers, especially women. Recently, ackee has emerged as high-priority species for domestication in Benin but information necessary to elaborate a clear domestication strategy is still very sketchy. This study addresses farmers' indigenous knowledge on uses, management and perception of variation of the species among different ethnic groups taking into account also gender differences.

**Methods:**

240 randomly selected persons (50% women) belonging to five different ethnic groups, 5 women active in the processing of ackee fruits and 6 traditional healers were surveyed with semi-structured interviews. Information collected refer mainly to the motivation of the respondents to conserve ackee trees in their land, the local uses, the perception of variation, the preference in fruits traits, the management practices to improve the production and regenerate ackee.

**Results:**

People have different interests on using ackee, variable knowledge on uses and management practices, and have reported nine differentiation criteria mainly related to the fruits. Ackee phenotypes with preferred fruit traits are perceived by local people to be more abundant in managed *in-situ *and cultivated stands than in unmanaged wild stands, suggesting that traditional management has initiated a domestication process. As many as 22 diseases have been reported to be healed with ackee. In general, indigenous knowledge about ackee varies among ethnic and gender groups.

**Conclusions:**

With the variation observed among ethnic groups and gender groups for indigenous knowledge and preference in fruits traits, a multiple breeding sampling strategy is recommended during germplasm collection and multiplication. This approach will promote sustainable use and conservation of ackee genetic resources.

## Background

Whether termed Non-Timber Forest Products (NTFPs) or designated as Agroforestry Tree Products (AFTPs) to differentiate between wild and domesticated products [1], many plants species are essential for the livelihoods of millions of poor farmers in tropical developing countries. They are part of the threatened biological assets of the rural poor representing an appreciable wealth of agrobiodiversity that has the potential to contribute to improve incomes, food security and nutrition. Local communities consider them essential elements not only in their diet but also in their food culture and rituals [2]. Unfortunately, these locally important species are often neglected leading to the erosion of their diversity and usefulness, further restricting development options for the poorest. Research to increase the value of these species and to make them more widely available would broaden the agricultural resource base and increase the livelihood options for rural communities.

Belonging to the Sapindaceae family, *B. sapida *(ackee in English) is a woody perennial multipurpose fruit tree species native to the Guinean forests of West Africa. The fleshy arils of the ripened fruits are edible. Seeds and capsules of the fruits are used for soap-making and for fishing, and all parts of the tree have medicinal properties. Fresh arils, dried arils and soap are traded in local and regional markets in Benin providing substantial revenues for farmers, especially women [3,4]. An economic survey conducted in 121 households in the rural township of Toukountouna (NW Benin) revealed that more than 9 tons of arils were produced in 2003 from which 80% were dried and traded in local markets generating more than US $ 10,000 of revenue. Interestingly, this revenue represents almost 20% of the family income competing with major staples such as maize (20%), sorghum (21%) and common beans & cowpeas (15%) [4]. *B. sapida *is widely cultivated in Jamaica where it had been introduced by slave traders during the 18^th ^century [5] with an annual turnover of approximately US $ 400 million in 2005 for the trade of the arils of the fruits[6].

Although largely overlooked by researchers in the region, the tree is highly valued by farmers and is an important component of traditional agroforestry systems in Benin. Recently, ackee has emerged as high-priority species for domestication in Benin after a national survey and ranking of Non-Timber Forest Products (NTFPs) [7]. General reasons to domesticate *B. sapida *are income generation, improvement of livelihoods strategies, satisfaction of farm household needs and agroecosystem diversification [3,7,8].

Tree domestication in agroforestry is defined as a farmer-driven and market-led process, which matches the intraspecific diversity of locally important trees to the needs of subsistence farmers, product markets, and agricultural environments. The first step before developing a domestication strategy for any species is to collate all available information on the species including botanic descriptions, geographic distribution, ecology, forest inventories, and farmers' survey, harvesting techniques, trade figures, conservation status and genetic variation patterns [1]. For *B. sapida*, some of these required key issues have been recently addressed [3,4,7-10]. Nevertheless, farmers' knowledge on uses, processing, management and perception about intraspecific variation are not yet fully documented. The documentation provides testable hypotheses for research that can accelerate the delivery of improved tree planting material to farmers [11]. This paper addresses these issues of farmer's indigenous knowledge and perception of variation of *B. sapida *at a national level considering different ethnic groups using the species and recognizing the potential gender differences.

## Methods

### Sampling

Previous works and early exploration have shown that *B. sapida *is distributed in different phytogeographic zones of Benin. Each phytogeographic zone hosts various ethnic groups and members of the same ethnic group are sometimes dispersed across different phytogeographic zones historically. However, even if people belonging to the same ethnic group are settled in different locations, they share together traditions, historical experiences, perceptions, values, attitudes, beliefs and language. Therefore, one may expect some variability on uses of natural resources and subsequent know-how not only among ethnic groups, but also among gender group.

**Figure 1:**
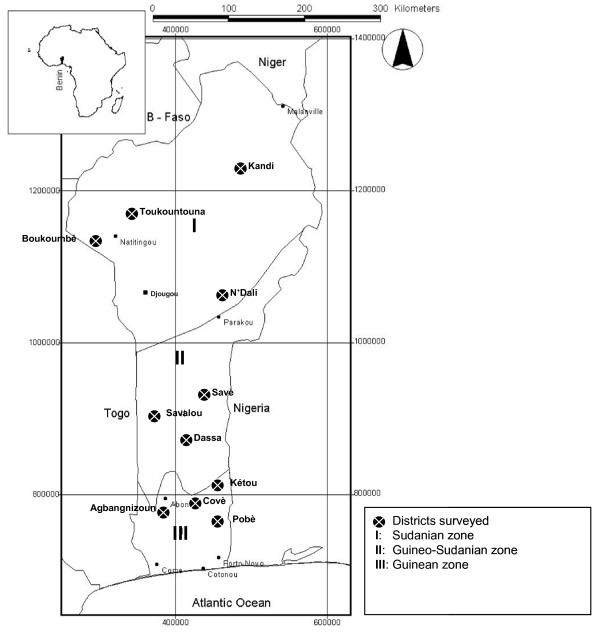
**Map of Benin showing the location of surveyed districts**.

According to the above-mentioned considerations and in order to get the maximum of information, eleven communes distributed in the three main phytogeographic zones (Figure 1) where *B. sapida *is known and used by local populations were included in the survey. In each commune, between 20 or 30 persons were randomly chosen. In total, 240 persons (50% women) belonging to the following Beninese ethnic groups (Adam and Boko, 1993): Batombu, Yoruba, Otamari, Natemba and Fon (Table 1). In addition, 5 women active in the processing of ackee fruits and 6 traditional healers were included.

**Table 1:** Common names of *Blighia sapida *in Benin

Ethnic group	Language	Local names
Batombu	Baatonu	Diremou
Yoruba	Nagot/Idatcha	Ichin/Iguichin
Otamari	Ditammari	Moufodom
Natemba	Naténi	Foulama
Fon	Fongbé/Mahi	Lissètin/Sissitin

### Ethnobotanical survey

Semi-structured interviews concerning the species were carried out once. Information's collected refer to the denomination of the species and its meaning, the motivation of the respondents to conserve ackee trees in their land use system, local uses, perception of variation, preferences in fruits traits, management practices to improve the production and regenerate ackee, the gender-specific tasks and responsibilities in the production and processing of ackee products.

The different traditional products obtained from ackee trees and inherent processing techniques were recorded. Likewise, the processing steps of each product, their variability and subsequent constraints were also recorded.

### Data analysis

Frequency distribution was used to compare answers within each ethnic group. The fidelity level (FL) [12] was calculated using the following formula: FL = Ip/Iu × 100%, where Ip is the number of informants answering positively on each question and Iu the total number of positive answers for each category of the questionnaire. This index was used to measure the consensus degree between informants and the relative importance of each category of knowledge within each ethnic group.

The interviewees were grouped according to ethnic group and gender group (men and women) so that in each ethnic group, two subgroups were defined: men (M) and women (F). With five ethnic groups, 10 subgroups were constituted. Because the size of subgroups differed and an interviewee could choose more than one ackee trait, the relative frequency of each trait was determined for each of the 10 subgroups. This parameter is defined as the proportion of interviewees belonging to the subgroup who identified the particular ackee trait. A data matrix comprising the relative frequencies of ackee traits was then submitted to Principal Component Analyses (PCA) using STATISTICA 8.0 [13]. This statistical method was used to identify traits that best explained the pattern of variation according to the different subgroups. For graphical purposes, the subgroups are labelled by preceding the ethnic group prefix (first three letters) with the label of one of the 10 subgroups defined above. For example, a man from Batombu ethnic group is labelled BatM, whereas a woman from the same ethnic group is labelled BatF.

Loglinear analysis was also performed using the PROC CATMOD available in SAS [14] with gender group (men or women) and ethnic group as dependent variables for each category of answer to detect possible association between people knowledge and their ethnic or gender membership.

## Results

### Local names of ackee

*B. sapida *is designated in each language by different local names shown in Table 1. The name *Foulama *used by the ethnic group Natemba means "groundnut of trees" by comparison of arils to nuts of peanut (*Arachis hypogaea *L.). All others local names do not have any particular meaning.

### Motivation to conserve ackee trees

In general, ackee trees are integrated in different land use systems across the country for a variety of reasons including the direct uses as food, soap, medicine, shade, myth and for its marketing value. Apart from the use as food, it was always the combination of two or three other reasons that determined the conservation of ackee in farmers' field. Table 2 shows the percentage of persons quoting each type of motivation in each ethnic group. The main motivation is always the use as food (between 53.3% among the Yoruba and 100% among the Otamari). The Otamari ethnic group showed also the highest motivation frequency for medicinal (73.3%) and marketing (36.7%) reasons. The Natemba is the second group using ackee for its marketing value. Natemba (40%) and Yoruba (30%) are the two ethnic groups valorising ackee soap while the tree provided shade for nearly 19% of the respondent in the Fon group.

**Table 2:** Variation in local knowledge of *Blighia sapida *according to five ethnic groups from Benin

Category/Criteria		Variant	Batombu (n = 40)			Yoruba (n = 60)			Otamari (n = 30)			Natemba (n = 30)			Fon (n = 80)			Total	
		
			*F*	%	FL	*F*	%	FL	*F*	%	FL	*F*	%	FL	*F*	%	FL	*F*	%
Motivation		Market	0	0.0	0.00	0	0.0	0.00	11	36.7	15.28	6	20.0	12.24	0	0.0	0.00	17	7.1
		
		Shade	3	7.5	6.12	0	0.0	0.00	0	0.0	0.00	1	3.3	2.04	15	18.8	20.83	19	7.9
		
		Medicine	15	37.5	30.61	12	20.0	26.67	22	73.3	30.56	6	20.0	12.24	3	3.8	4.17	58	24.2
		
		Soap	0	0.0	0.00	1	1.7	2.22	9	30.0	12.50	12	40.0	24.49	2	2.5	2.78	24	10.0
		
		Food	28	70.0	57.14	32	53.3	71.11	30	100.0	41.67	24	80.0	48.98	51	63.8	70.83	165	68.8
		
		Myth	3	7.5	6.12	0	0.0	0.00	0	0.0	0.00	0	0.0	0.00	1	1.3	1.39	4	1.7
		
		Σ*F*	49	-	-	45	-	-	72	-	-	49	-	-	72	-	-	-	-

Uses	*Food*	Fresh aril	29	72.5	19.59	27	45.0	42.19	25	83.3	20.49	22	73.3	22.22	51	63.8	72.86	154	64.2
		
		Dried aril	29	72.5	19.59	21	35.0	32.81	25	83.3	20.49	22	73.3	22.22	3	3.8	4.29	100	41.7
		
		Fried aril	29	72.5	19.59	3	5.0	4.69	1	3.3	0.82	6	20.0	6.06	0	0.0	0.00	39	16.3
		
		Boiled aril	29	72.5	19.59	10	16.7	15.63	25	83.3	20.49	21	70.0	21.21	8	10.0	11.43	93	38.8
		
		Vegetable	0	0.0	0.00	0	0.0	0.00	0	0.0	0.00	3	10.0	3.03	0	0.0	0.00	3	1.3
	
	*Fisheries*	Fisheries	1	2.5	0.68	0	0.0	0.00	22	73.3	18.03	10	33.3	10.10	0	0.0	0.00	33	13.8
	
	*Soap*	Soap	23	57.5	15.54	1	1.7	1.56	19	63.3	15.57	15	50.0	15.15	6	7.5	8.57	64	26.7
		
		Capsule to wash	7	17.5	4.73	2	3.3	3.13	4	13.3	3.28	0	0.0	0.00	2	2.5	2.86	15	6.3
	
	*Repellent*	Repellent	1	2.5	0.68	0	0.0	0.00	1	3.3	0.82	0	0.0	0.00	0	0.0	0.00	2	0.8
		
		Σ*F*	148	-	-	64	-	-	122	-	-	99	-	-	70	-	-	-	-

Variation in fruits traits	*Differentiation in fruits traits*	Fruit size	29	72.5	38.67	13	21.7	39.39	21	70.0	33.87	20	66.7	42.55	5	6.3	45.45	88	36.7
		
		Fruit shape	1	2.5	1.33	1	1.7	3.03	1	3.3	1.61	0	0.0	0.00	0	0.0	0.00	3	1.3
		
		Aril colour	1	2.5	1.33	2	3.3	6.06	0	0.0	0.00	2	6.7	4.26	0	0.0	0.00	5	2.1
		
		Aril size	1	2.5	1.33	1	1.7	3.03	1	3.3	1.61	0	0.0	0.00	0	0.0	0.00	3	1.3
		
		Aril taste	3	7.5	4.00	4	6.7	12.12	5	16.7	8.06	1	3.3	2.13	0	0.0	0.00	13	5.4
		
		Seed size	12	30.0	16.00	1	1.7	3.03	12	40.0	19.35	5	16.7	10.64	1	1.3	9.09	31	12.9
	
	*Preference in fruits traits*	Fruit size	28	70.0	37.33	9	15.0	27.27	19	63.3	30.65	0	0.0	0.00	0	0.0	0.00	56	23.3
		
		Fruit shape	0	0.0	0.00	1	1.7	3.03	1	3.3	1.61	0	0.0	0.00	0	0.0	0.00	2	0.8
		
		Aril colour	0	0.0	0.00	0	0.0	0.00	1	3.3	1.61	19	63.3	40.43	5	6.3	45.45	25	10.4
		
		Aril size	0	0.0	0.00	1	1.7	3.03	1	3.3	1.61	0	0.0	0.00	0	0.0	0.00	2	0.8
		
		Aril taste	0	0.0	0.00	4	6.7	10.26	6	20.0	10.00	1	3.3	1.79	1	1.3	1.96	12	5.0
		
		Σ*F*	75	-	-	33	-	-	62	-	-	47	-	-	11	-	-	-	-

Propagation and regeneration practices		Assisted tree regeneration	18	45.0	24.00	2	3.3	5.13	17	56.7	28.33	19	63.3	33.93	5	6.3	9.80	61	25.4
		Transplanting	27	67.5	36.00	25	41.7	64.10	19	63.3	31.67	21	70.0	37.50	40	50.0	78.43	132	55.0
		Sowing	30	75.0	40.00	5	8.3	12.82	16	53.3	26.67	15	50.0	26.79	5	6.3	9.80	71	29.6
		Σ*F*	75	-	-	39	-	-	60	-	-	56	-	-	51	-	-		

Management practices to improve production		Ringing	0	0.0	0.00	3	5.0	7.69	2	6.7	3.33	0	0.0	0.00	0	0.0	0.00	5	2.1
		Grazing protection	1	2.5	1.23	1	1.7	3.70	1	3.3	1.67	1	3.3	1.92	0	0.0	0.00	4	1.7
		Tree/crop association	8	20.0	9.88	10	16.7	37.04	3	10.0	5.00	4	13.3	7.69	17	21.3	44.74	42	17.5
		Pruning	25	62.5	30.86	4	6.7	14.81	20	66.7	33.33	22	73.3	42.31	1	1.3	2.63	72	30.0
		Fire protection	27	67.5	33.33	12	20.0	44.44	22	73.3	36.67	16	53.3	30.77	20	25.0	52.63	97	40.4
		Mulching/	20	50.0	24.69	0	0.0	0.00	14	46.7	23.33	9	30.0	17.31	0	0.0	0.00	43	17.9
		Σ*F*	81	-	-	27	-	-	60	-	-	52	-	-	38	-	-	-	-

n = number of interviewees, *F *= Frequency of answer, Σ*F *= total number of positive answer per ethnic group, FL = Fidelity Level

In addition, women conserve ackee for soap making and its commercial value, while men keep them for shade. The trade of ackee products seems to be restricted to the ethnic groups Otamari and Natemba. The motivation to conserve ackee trees varied significantly among ethnic groups (χ^2 ^= 14.49, df = 4, p < 0.01) but not among gender group (Table 3). From one ethnic group to the other the motivation depended on the gender (χ^2 ^= 13.11, df = 4, p < 0.05).

**Table 3:** Results of log linear analysis between indigenous knowledge and traditional management variables, and ethnic group membership and gender of the respondent

Indigenous knowledge and traditional management variables	Source of variation	Degree of freedom	Chi-Square	*P *value
Motivation to conserve	EG	4	14.49	< 0.01
	GG	1	0.10	0.754
	EG*GG	4	13.11	< 0.05
	Likelihood ratio	25	164.85	< 0.001
Uses as Food	EG	4	11.37	< 0.05
	GG	1	5.12	< 0.05
	EG*GG	4	8.23	0.084
	Likelihood ratio	28	157.48	< 0.001
Uses as Soap	EG	4	18.09	< 0.01
	GG	1	7.30	< 0.01
	EG*GG	4	5.58	0.232
	Likelihood ratio	16	104.24	< 0.001
Uses in Fisheries	EG	4	55.98	< 0.001
	GG	1	1.87	0.172
	EG*GG	4	0.89	0.926
	Likelihood ratio	5	37.48	< 0.001
Differentiation in fruits traits	EG	4	9.54	< 0.05
	GG	1	0.30	0.586
	EG*GG	4	3.03	0.553
	Likelihood ratio	13	82.46	< 0.001
Preference in fruits traits	EG	4	31.91	< 0.001
	GG	1	1.24	0.266
	EG*GG	4	3.92	0.417
	Likelihood ratio	12	39.18	< 0.001
Propagation and Regeneration practices	EG	4	5.84	0.212
	GG	1	3.93	< 0.05
	EG*GG	4	11.37	< 0.05
	Likelihood ratio	19	77.04	< 0.001
Management Practices to improve production	EG	4	14.21	< 0.01
	GG	1	7.95	< 0.01
	EG*GG	4	12.50	< 0.05
	Likelihood ratio	32	99.78	< 0.001

EG: Ethnic group, G: Gender, EG*GG: Interaction between ethnic group and gender group

The fidelity level (FL) of motivation highlighted the uses as food, medicine, soap and the commercial value as the most important (Table 2).

### Main uses, post-harvest handling and processing of ackee

#### Use of ackee as food

At maturity, arils are consumed directly fresh, added to sauce to replace sesame (*Sesamum indicum *L.) seeds or peanuts (*Arachis hypogaea *L.), or grounded into powder and added to the sauce mainly to release its oil contents. Arils are also fried in peanut (*A. hypogaea*) or oil palm (*Elaeis guineensis *Jacq.) oil. It can be parboiled with salt and sometimes spices. Arils are dried mainly for conservation purpose and this is usually the commercialized form at local markets and/or for shipment toward cities. For drying, arils are exposed to the sun during 4 days and thereafter it can be stored for 2 weeks. The dried arils can be used as described above in the fresh, boiled or fried forms. Young leaves may be parboiled and used like any other African leafy vegetables.

The main difficulty highlighted by nearly 70% respondents is the long-term storage of arils. The absence of efficient drying techniques makes the storage of large quantities of arils difficult, especially when fruits mature in the rainy season. Roads are usually degraded at that time of the year, making transport of the production toward markets in big cities difficult. This results in the loss of a large part of the production due to destruction by insects or birds when mature fruits are abandoned on trees.

The FL revealed that there was a high consensus between informants for fresh aril in all ethnic groups (between 19.6 and 72.9), for boiled aril and dried aril in Otamari, Natemba and Batombu, the latter ethnic group also for fried aril (Table 2). Fresh, dried and boiled arils showed the same and high (more than 70%) use frequency within the Batombu, Otamari and Natemba communities. The use of leaves as vegetable is restricted to the ethnic group Natemba (10%). People belonging to the Fon ethnic group had a high preference to the fresh aril (63.8%) and only few persons favor the other form of food use. Significant differences were detected for the use of ackee as food according to the ethnic group (χ^2 ^= 11.37, df = 4, p < 0.05) and the gender of the respondents (χ^2 ^= 5.12, df = 4, p < 0.05) (Table 3).

#### Use of ackee as soap

Capsules of the fruits have the property of producing saponins, which lather in water and are used for washing. In the Pobè region (South-East Benin), it is rather the whole immature fruits that are cut in small pieces and plunged into water for washing clothes. According to the interviewees, this type of utilization was very popular in the past across the country before the introduction of manufactured soap. Today the use of fruit capsules as soap is practiced mainly in the Batombu (17.5%) and Otamari (13.3%) ethnic groups, and the associated FL were fairly low (Table 2).

**Figure 2:**
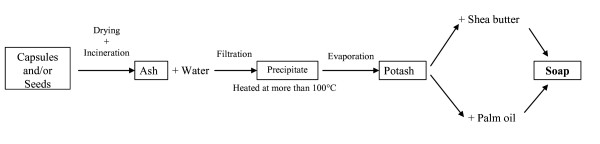
**Manufacturing process of *Blighia sapida *soap in Benin**.

The manufacturing process of ackee soap is shown in Figure 2. In the saponification process, shea [(*Vitellaria paradoxa *C.F.Gaertn.)] butter can be substituted by palm oil depending on the availability. Shea butter is widespread in the Northern part of Benin and palm oil in the South. Nowadays, ackee soap is mainly produced and commercialized by women from the ethnic groups Otamari (63.3%), Batombu (57.5%) and Natemba (50%). The soap is valued mainly for its medicinal and esthetical properties (Table 4).

**Table 4:** Therapeutic virtues and/or properties of *Blighia sapida *soap in Benin

Virtue and medicinal properties	Number of quotations	
	
	♂	♀
Scabies	24	20
Tinea	22	19
Antipyretic	20	23
Antiseptic, dermatosis	23	27
Softening of the skin	20	29
Washing	3	6
Burns	21	24

Loglinear analysis showed significance for the use of ackee as soap among ethnic group (χ^2 ^= 18.09, df = 4, p < 0.01) and among gender (χ^2 ^= 7.30, df = 1, p < 0.01).

#### Use of ackee in fisheries

The bark, seeds and capsules are dried, reduced into powder and used to poison fishes so that they are rendered easier to catch. This type of utilization is exclusively restricted to the ethnic groups Otamari and Natemba located in the North-West of Benin. 80% of men and 90% of women have knowledge about this use. In Boukoumbé where there is no river for fishing, capsules and bark are sold or exchanged against fishes with fishermen from other villages. The use of ackee in fisheries differed significantly between ethnic groups (χ^2 ^= 17.02, df = 4, p < 0.001) and by gender (χ^2 ^= 6.01, df = 4, p < 0.05).

#### Use of ackee as repellent

The spreading of ashes obtained from calcined capsules is a repellent for some insect pest to cultures such as cowpea (*Vigna unguiculata *(L.) Walp.) or common bean *Phaseolus vulgaris *(L.) in the region of N'Dali (North-East Benin). In Boukoumbe, the bark is first dried, then crushed and afterwards mixed with seeds of pearl millet (*Pennisetum glaucum *(L.) R.Br.) and African finger millet (*Eleusine coracana *(L.) Gaertn. ssp. *africana *(Kennedy & O'Byrne) Hilu & de Wet) before sowing to avoid insects' attacks. However, only 4 male interviewees have mentioned this type of use.

#### Traditional medicinal uses of ackee

In total, 22 diseases have been recognized to be healed with ackee. Dental decay, fever, malaria, internal haemorrhage, dysentery, burns, eyes inflammation, yellow fever, constipation, cutaneous infections, whitlow and head lice are the most common. All parts (bark, capsules, seeds, roots, leaves) are involved in the composition of drugs (Table 5). The bark is useful in the treatment of 13 different diseases followed in decreasing order by leaves (8), capsules (3), roots and seeds (2). This type of knowledge is kept mostly by old people and traditional healers in the communities and varied sometimes from one ethnic group to the other.

**Table 5:** Medicinal properties of *Blighia sapida *in Benin

N°	Disease/virtue	Composition/preparation	Dosage
1	Whitlow	Bark + common beans or cowpeas + salt. Crush the mixed	Application of the mixture on the finger
		Crush roasted seeds + palm kernel oil	
		Crush roasted seeds roasted and + palm oil	
		Incinerate a mix of ackee seeds + cashew nuts. Add palm oil to the ashes	
2	Head lice	Incinerate the capsules to obtain ashes	Use the ashes to wash the head
3	Dental decay	Crush seeds + salt	Put on the decaying teeth
		Crush dried bark	Put on the hole of the decaying teeth
4	Child Fever	Infusion of the roots	Wash the child with the infusion
		Decoction of leaves and bark	Wash the child with the decoction
		Triturate leaves with water	
5	Fever	Triturate leaves of ackee and teak (*Tectona grandis *L. f.)	
		Triturate leaves of ackee and mango (*Mangifera indica *L.)	
6	Yellow Fever	Crush dried bark into powder + salt	Add the mix to a porridge and drink it
		Crush bark + African locust bean (*Parkia biglobosa *(Jacq.) R.Br. ex G.Don) mustard	Eat
7	Eyes problems	Soak bark in water	Wash the eyes with the water
8	Bite of scorpion or snake	Crush dried bark into powder + salt	Application on the wounded zone and eat also
9	Malaria	Infusion of bark + seeds of green pepper (*Capsicum annuum *L.) + soya bean (*Glycine max *(L.) Merr.) leaves	Take three glass per day
		Infusion of ackee and papaya (*Carica papaya *L.) leaves	
		Infusion of bark	
		Decoction of leaves	
		Decoction of dried bark	
10	Healing of wound	Crush bark or seeds into powder	Application into the wound
11	Apparition of the first children's teeth	Decoction of leaves and bark	Make drink the child
12	Abscess	Crush bark + common beans or cowpeas	Application on the abscess
		Crush roasted seeds + oil palm (*Elaeis guineensis *Jacq.) oil	
13	Burns	Crush and press the bark to gather the juice + honey	Application on the burn area
14	Cutaneous infections, buttons on the body	Infusion of leaves and bark	Take a shower with the infusion
15	Internal hemorrhage	Crush dried bark	Add to porridge and drink
16	Pregnant woman blood flow	Macerate leaves previously exposed to the dew + limestone	Drink three glasses per day
17	Constipation	Decoction of bark	
18	Anemia	Decoction of roots	
19	Vomiting	Decoction of leaves	
20	Dysentery	Decoction of leaves + shea (*Vitellaria paradoxa *C.F. Gaertn.) butter	
21	Guinea worm infection (Dracunculiasis)	Crush dried bark + shea (*Vitellaria paradoxa *C.F. Gaertn.) butter + potash	Apply the mix on the skin
22	Fracture	Macerate leaves	Massage the fractured limb

### Perception of variation and preferences in ackee fruit traits

#### Existence of different types of ackee

Nine criteria were reported to characterize different types of ackee from which seven are related to the fruit and its different parts. Fruit size is by far the most quoted criterion followed by aril taste, size and colour of aril, and seed size (Table 6). According to farmers, fruit size is positively correlated with aril size.

**Table 6:** Perception of variation of *Blighia sapida *by local people in Benin

Differentiation criteria	Different type reported	Characteristic	Percent of interviewees
Size fruit	Small	Narrow leaflets, wild tree, small aril	36.67
	Large	Larger leaflets, planted tree, large aril	
Aril taste	Soft	-	5.42
	Hard	-	
Aril size + seed size	Large aril and small seed	-	2.08
	Small aril and large seed	-	
Aril colour	Light yellow	Less tasty and hard to conserve	2.08
	Yellow	Tasty and easy to conserve	
Fruit shape	Elongate	-	1.25
	Short	-	
Fruit size + fruit shape	Small and elongate	Aril very tasty	1.25
Height of the tree before the first fructification	Small	Between 1.5 and 2 meters	1.25
	Tall	More than 2 meters	
Capsule's number of chambers	3 chambers		0.83
	4 chambers		
	5 chambers		
Leaflets width	Large	-	0.83
	Narrow	-	

#### Differentiation and preferences in ackee fruit traits

The Fon appeared to have just residual knowledge about fruits traits. Indeed, only 7.5% could differentiate ackee based on fruit size, while this frequency varied between 21.7% (Yoruba) and 72.5% (Batombu). Seed size was the second important criterion and it followed the same tendency as observed for fruit size. Aril taste was relatively an important criterion of differentiation for the Otamari (16.7%) and the others criteria were minor (Table 2).

Preferred fruits traits were the same in which local population perceived variation in fruits traits. The fruit size was the most important criterion among the Yoruba (15.0%), Otamari (63.3%) and Batombu (70.0%) communities. Aril color was very important for the Natemba (63.3%) while aril taste was relevant for the Otamari (16.7%). Farmers indicated that managed trees exhibited their preferred traits more frequently than trees in the wild and/or unmanaged trees. There were significant differences for differentiation (χ^2 ^= 9.54, df = 4, p < 0.05) and preference (χ^2 ^= 31.91, df = 4, p < 0.001) in fruit traits among ethnic groups.

The FL of differentiation in fruit traits highlighted the importance of fruit size in all ethnic groups and for seed size in the Batombu and Otamari. With preference in fruits traits, FL revealed the importance of fruit size with the Batombu, Otamari and Yoruba, and color of aril for the Natemba and Fon ethnic groups.

### Traditional management practices

#### Propagation and indigenous regeneration practices

Three regeneration techniques of ackee were recorded: sowing, transplanting and assisted tree regeneration. The reason behind each regeneration method and the practical implementation are summarized in Table 7. Transplanting of wildings was the most important regeneration method at the national level followed by sowing and assisted tree regeneration. Women seemed to practice more often sowing than men. Sowing was more common in the ethnic groups Batombu (75.0%), Otamari (63.1%) and Natemba (70%) and is almost as important as transplanting and assisted tree regeneration. Assisted tree regeneration was mainly practiced by the Batombu (45.0%), Otamari (56.7%) and Natemba (63.3%). The FL confirmed the importance of those practices in every community except the Yoruba and Natemba for sowing and assisted tree regeneration (Table 2). Significant differences were detected for this type of knowledge according to gender (χ^2 ^= 3.93, df = 4, p < 0.05) and the interaction between gender group and ethnic group (χ^2 ^= 11.37, df = 4, p < 0.05).

**Table 7:** Propagation, regeneration and management practices of *Blighia sapida *in Benin

Practice		Reason/function	Implementation
Propagation and regeneration practices	Assisted tree regeneration	Favour natural regeneration	Young plants are staked to be easily visible and protected from tillage, grazing and fire
	Transplanting of wildings	Use of naturally regenerated seedlings and saplings	Seedlings and saplings are removed and replanted in an appropriate area and given essential care
	Sowing	Multiply the best provenance with the preferred fruits traits	Seeds from the most vigorous or best fruit yielding trees are selected and put together. After germination during the rainy season, they are transplanted in an appropriate location to receive care
Management practices to improve production	Ringing	Stimulate fruit production	A shallow 10 cm-wide ring of bark is cut from the trunk at breast height just before flowering
	Grazing protection	Avoid destruction of seedlings and saplings by domestic animals	Establish fence of cacti or rocks around the seedlings and saplings
	Tree/crop association	Diversification, soil protection, shadow for cultures, creation of microclimate favourable for crops	To leave naturally growing or planted ackee trees in farmland and to plant crops such as millet, sorghum. maize, yam in the same field
	Pruning	Improved fruit production, reduction of shade on understorey crops, firewood	Cutting back certain branches
	Fire protection	Avoid fire damages to trees that affect fruit yield and destroy seedlings and saplings	Tillage, weeding and clearing around the seedlings, saplings and trees
	Mulching/organic fertilization	Rapid growth of seedlings and saplings and increasing fruit production	Leaf mulch, animal manure, compost and crop residues near the root and sprinkling with water

#### Traditional management practices to improve the production

Pruning, ringing, protection from grazing, tree/crop association, fire protection and mulching are the management practices used by farmers to improve production (Table 7). Fire protection was the most important practice in all ethnic groups confirmed by the high FL value. In addition, pruning and mulching were very important for the Batombu, Otamari and Natemba (Table 2). Significant differences were detected among ethnic groups (χ^2 ^= 14.21, df = 4, p < 0.01), among gender (χ^2 ^= 7.95, df = 1, p < 0.01) and for the interaction between gender group and ethnic group (χ^2 ^= 12.50, df = 4, p < 0.05).

### Links between indigenous knowledge, perception of variation and traditional management of ackee in Benin

**Figure 3:**
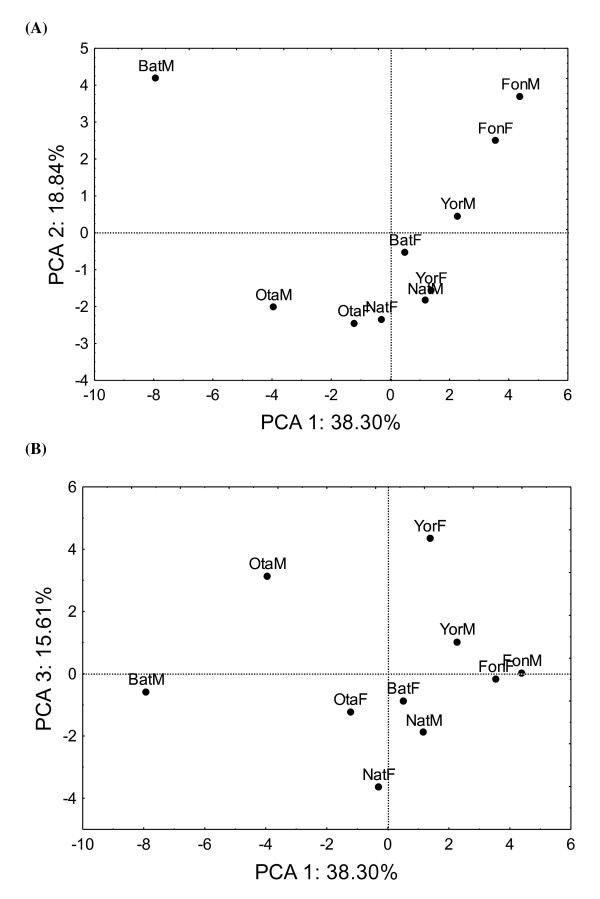
**Projection of ethnic groups and gender into the principal component analysis (PCA) axes**. (A) PCA axes 1 and 2, (B) PCA axes 1 and 3.

The result of the principal component analysis (PCA) performed on the indigenous knowledge, the perception of variation and the traditional management of ackee showed that the first three axes explained 72.8% of the variation observed. Therefore, only the first three axes were used to describe the relationship between people's knowledge of the species and their ethnic group and gender. Table 8 shows the sign of correlation values between the different criteria and the three PCA axes. Figure 3A and 3B shows the projection of the different ethnic/gender groups onto the first and second, first and third axes respectively.

**Table 8:** Correlation between *Blighia sapida *characteristics and principal component analysis (PCA) factors

Category/Criteria	Variant	PCA 1	PCA 2	PCA 3
Motivation	Market	-0.236	**-0.611**	-0.353
	Shade	0.303	**0.676**	-0.068
	Medicine	**-0.833**	-0.163	0.343
	Soap	-0.117	**-0.612**	**-0.509**
	Food	0.204	**0.763**	0.039
	Myth	**-0.634**	**0.673**	-0.098
Food use	Fresh aril	0.218	**0.862**	-0.033
	Dried aril	**-0.903**	-0.224	0.002
	Fried aril	**-0.708**	0.435	-0.240
	Boiled aril	**-0.866**	0.012	-0.363
	Vegetable	-0.027	-0.323	**-0.54**9
Fisheries use	-	-0.375	**-0.610**	-0.042
Soap use	Soap	**-0.898**	0.046	-0.320
	Capsule to wash	**-0.811**	0.467	0.222
Use as repellent	-	**-0.853**	0.221	0.284
Perception of variation	Size fruit	**-0.925**	-0.008	-0.240
	Fruit shape	**-0.657**	0.052	**0.674**
	Color aril	-0.181	-0.194	0.168
	Size aril	**-0.657**	0.052	**0.674**
	Taste aril	**-0.720**	-0.080	**0.552**
	Size seed	**-0.907**	-0.024	-0.207
Preference in fruits traits	Size fruit	**-0.844**	0.174	0.133
	Fruit shape	-0.183	-0.369	**0.840**
	Color aril	0.213	-0.300	**-0.684**
	Size aril	-0.183	-0.369	**0.840**
	Taste aril	-0.154	-0.458	**0.616**
Propagation and regeneration practices	Assisted tree regeneration	**-0.795**	-0.082	-0.463
	Transplanting of wildings	0.139	**0.889**	0.054
	Sowing	**-0.885**	0.299	-0.305
Management practices to improve production	Ringing	0.053	-0.140	0.241
	Grazing protection	0.003	-0.332	0.124
	Tree/crop association	0.434	**0.778**	0.184
	Pruning	**-0.880**	-0.100	-0.382
	Fire protection	**-0.725**	**0.562**	-0.069
	Mulching/organic fertilization	**-0.891**	-0.034	-0.181

It can be deduced from results shown in Table 5 and Figure 3 that the male Batombu and Otamari (BatM and OtaM) are motivated to conserve ackee on their land for its utility as medicine and myth; use of the dried, boiled or fried aril; favour the use of the soap and as repellent; they perceived variation in fruit size and shape, seed size and aril size and taste; selected fruits based on their size; practiced sowing, assisted tree regeneration, used ackee for mulching and organic fertilization, and practiced pruning and fire protection. The criteria mentioned above are all relevant also for the Fon (men and women) with the following additional ones: market, shade, soap, food and myth in the motivation category; use in fishery; transplantation and tree/crop association (Table 8). The PCA 2 axe clearly separated also the Otamari and Natemba women groups. The Natemba women, the Yoruba women and the Otamari men are highly correlated with the PCA 3 that is explained by the motivation to conserve ackee tree for its utility as soap, the use of the leaves as vegetable, the perception of variation in fruit shape, aril size and aril taste, and the following preferences in ackee fruits traits: fruit shape, aril colour, aril size and aril taste.

## Discussion and conclusions

### Indigenous knowledge and valorisation of ackee products

*B. sapida *is well known in Benin. It has been utilized for centuries and is still an important plant genetic resource today. Each ethnic group has different names for the species indicating age-old knowledge and uses. In general, by providing different products, services and having a commercial value, ackee is conserved for its multipurpose properties.

Apart from the use as vegetable in the Natemba ethnic group mentioned above, others utilizations of the aril have been reported also in West Africa [15-18] and in the Caribbean [19,20]. Farmers managed the species firstly to meet their own needs and also for the commercial value of arils and soap [3,4].

Future researches on the technological characteristics of the fruits and the design of small storage techniques are needed to help farmers to improve the conservation and long-term storage of arils. The best way to do that would be to establish small processing units managed by farmers in villages or at the communal level. The exact knowledge of composition is the basis for any successful technological process [21]. Preliminary analyses of the physical composition of arils from Toukountouna (North-West Benin) have shown that it includes 46% of oil, 47% of fibres and 3% of proteins [10]. The food value of 100 g of raw arils from Mexico is as follows: Moisture (57.60 g), Protein (8.75 g), Fat (18.78 g), Fibre (3.45 g), Carbohydrates (9.55 g), Ash (1.87 g), Calcium (83 mg), Phosphorus (98 mg), Iron (5.52 mg), Thiamine (0.10 mg), Riboflavin (0.18 mg), Niacin (3.74 mg) and Ascorbic Acid (65 mg) [19].

Characterization of ackee seed oil and defatted cake of seeds from Southern Benin compared to seeds from Nigeria and Côte d'Ivoire revealed differences in chemical properties and composition of seeds oils (saponification value, iodine value, oleic acidity, peroxide value); fatty acid composition, proximate composition (moisture, fat, crude fibre, total sugars, starch, proteins) and mineral composition (K, N, P, Ca, Mg, Na, Cu, Zn, Mn, Fe, Fe, Ash) and 17 amino acids [22-24]. Those results highlighted the potential of ackee seeds for industrial use as lubricants, surfactants and as oil for consumption that should be further explored. Feeding trial experiments conducted in savannas areas of Nigeria have shown that ackee leaves are good fodder resource for West African Dwarf goats especially in the dry season [25]. This is probably good news for animal breeders in the region because ackee trees flush during dry seasons in many part of West Africa when the availability of grasses to feed ruminants decreases drastically.

Some of the medicinal values attributed to ackee in Benin are known in other countries where the species occurs as well. The bark is used in Ivory Coast together with some spices to relieve pains; leaves and barks are used in association to treat sore stomach, epilepsy and yellow fever in Columbia [26]. In Ghana, the bark is one of the ingredients used in a concoction administered for epilepsy; leave juice is used for washing or as drops for sore eyes, conjunctivitis and trachoma; the pulp of twiggy leaves is applied on the forehead to treat migraine/headache [26]. *B. sapida *is also a natural source of carboxycyclopropylglycine used in pharmacy. The extraction of this non-proteinogenic amino acid from ackee offers the possibility of avoiding the expensive synthetic procedures [27]. Furthermore, *B. sapida *has antidiabetic activity [28]. The use of this important traditional medicinal knowledge in a rational way remains a challenge to modern scientific disciplines such as pharmacology. More research is necessary to analyze the properties and therapeutic virtues attributed to the soap as a preliminary step to the mechanization of the production. The confirmation of the virtues of ackee soap can boost its production and contribute to a better valorisation of the enormous quantity of capsules and seeds that are usually thrown away.

Ackee is not well known in Benin for its utility as repellent. However, experiments conducted in Trinidad and Tobago had shown that other fruit parts (epidermis, aril and seed) have repellent properties against stored-product insect pests, namely, *Callosobruchus maculatus*, *Cryptolestes ferrugineus*, *Tribolium castaneum *and *Sitophilus zeamais *[29,30].

### Traditional management and domestication of ackee

The various differentiation criteria of ackee trees mentioned above calls for an appropriate characterization to investigate the existence of eventual varieties, and/or to detect the effect of the ongoing domestication process practiced by farmers in their different land-use systems. For instance, the positive correlation reported between fruit size and aril size needs to be tested by morphometric study to characterize the diversity of ackee fruits traits.

All traditional silvicultural management practices to improve the production were reported for other important agroforestry parkland species such as *Vitellaria paradoxa *and *Parkia biglobosa *(Jacq.) R.Br. ex Benth. in West Africa [31]. There is a consensus among local people that these management practices favour the abundance of better phenotypes, in this case fruits with preferred traits. This link between management techniques and the perception of variation suggests that there is selection going on with a tendency to increase phenotypes producing desired fruits in managed populations. However, reasons for the "superior" perception of trees under management need further analyses. It is particularly challenging and important to distinguish genetic from environment impacts on phenotypes.

Evidence that farmers have domesticated other African indigenous trees has been reported for *Dacryodes edulis *H.J. Lam and *Irvingia gabonensis *(Aubry-Lecomte ex O'Rorke) Baill. [32-34]; *Vitellaria paradoxa *C.F. Gaertn. [35] and *Sclerocarya birrea *(A. Rich.) Hochst. subsp. *caffra (Sond.) *Kokwaro[36]. Processes of plants domestication associated to silvicultural management were also documented for many species in the Tehuacán Valley of Mexico including *Stenocereus stellatus *(Pfeiff.) Riccob. [37] and *Ceiba aesculifolia *(Kunth) Britten & Baker [38].

### Implications for improvement and conservation of ackee genetic resources

In general, the ethnobotanical survey revealed clearly that indigenous knowledge about ackee varies according to ethnic group and gender. Particularly three ethnic groups (Batombu, Otamari and Natemba) had a great knowledge about the species. In addition, the multivariate analysis showed also clearly the separation between the knowledge of men and women within the Otamari, Batombu and Yoruba communities.

Selection or breeding programs should focus on ackee trees with preferred traits important for local populations. For instance when looking at preferred fruit traits, this study showed that ackee fruit size is the most important trait for men (Batombu, Otamari and Yoruba). Fruit shape, aril colour, aril size and aril taste were the preferred fruit traits for Batombu women, Otamari men and all Yoruba and Natemba. Those differences needs to be taken into account in any research/development programs related to germplasm sampling and ackee improvement.

Domestication can reduce the genetic diversity of wild populations if cultivars replace autochthonous populations on a large scale. It can also increase the level of variability at desired traits in semi-domesticated populations suggesting that varieties may have multiple origins [1,34]. The effect of the artificial selection reported in this study on the genetic diversity and structure of ackee is not yet known and needs to be evaluated properly. This prerequisite is essential to avoid that the intensification of the domestication process will lead to a progressive elimination of individuals with non-desired quality, and a subsequent loss of genetic diversity.

Conservation of ackee genetic resources can be done effectively through cultivation of the species in agroforestry systems, its maintenance on protected areas where they occurs and maintenance of seeds in gene banks. Since preferred traits vary among ethnic groups and gender, the strategy should be specific and should target not only the morphotypes recognized by local populations but should also integrate the population genetics information.

## Competing interests

The authors declare that they have no competing interests.

## Authors' contributions

EMRM designed and performed the field work, analyzed and wrote the draft. SB and E-MO gave technical support and conceptual advices. FR supervised the work and improved the manuscript.

All authors have read and approved the final manuscript.
